# Discovery of New Potent anti-MERS CoV Fusion Inhibitors

**DOI:** 10.3389/fphar.2021.685161

**Published:** 2021-06-02

**Authors:** Mahmoud Kandeel, Mizuki Yamamoto, Byoung Kwon Park, Abdulla Al-Taher, Aya Watanabe, Jin Gohda, Yasushi Kawaguchi, Kentaro Oh-hashi, Hyung-Joo Kwon, Jun-ichiro Inoue

**Affiliations:** ^1^ Department of Biomedical Sciences, College of Veterinary Medicine, King Faisal University, Al-Ahsa, Saudi Arabia; ^2^ Department of Pharmacology, Faculty of Veterinary Medicine, Kafrelsheikh University, Kafrelsheikh, Egypt; ^3^ Research Center for Asian Infectious Diseases, Institute of Medical Science, The University of Tokyo, Tokyo, Japan; ^4^ Division of Cellular and Molecular Biology, Department of Cancer Biology, Institute of Medical Science, University of Tokyo, Tokyo, Japan; ^5^ Department of Microbiology, Hallym University College of Medicine, Chuncheon, South Korea; ^6^ Division of Molecular Virology, Department of Microbiology and Immunology, The Institute of Medical Science, The University of Tokyo, Tokyo, Japan; ^7^ Department of Chemistry and Biomolecular Science, Faculty of Engineering, Gifu University, Gifu, Japan; ^8^ Senior Professor Office, The University of Tokyo, Tokyo, Japan

**Keywords:** coronavirus, MERS-CoV, fusion inhibitors, antivirals, drug discovery

## Abstract

Middle East respiratory syndrome coronavirus (MERS-CoV), capable of zoonotic transmission, has been associated with emerging viral pneumonia in humans. In this study, a set of highly potent peptides were designed to prevent MERS-CoV fusion through competition with heptad repeat domain 2 (HR2) at its HR1 binding site. We designed eleven peptides with stronger estimated HR1 binding affinities than the wild-type peptide to prevent viral fusion with the cell membrane. Eight peptides showed strong inhibition of spike-mediated MERS-CoV cell-cell fusion with IC50 values in the nanomolar range (0.25–2.3 µM). Peptides #4–6 inhibited 95–98.3% of MERS-CoV plaque formation. Notably, peptide four showed strong inhibition of MERS-CoV plaques formation with EC50 = 0.302 µM. All peptides demonstrated safe profiles without cytotoxicity up to a concentration of 10 μM, and this cellular safety, combined with their anti-MERS-CoV antiviral activity, indicate all peptides can be regarded as potential promising antiviral agents.

## Introduction

The Middle East respiratory syndrome coronavirus (MERS-CoV) causes severe respiratory manifestations, including fever, persistent cough, and pneumonia, with occasional gastrointestinal symptoms such as vomiting, diarrhea, and death from renal failure (Assiri et al., 2013; Nassar et al., 2018). MERS-CoV is fatal for approximately one-third of people infected (Ahmadzadeh et al., 2020), which is regarded as a high fatality rate.

There is no vaccine or drug currently approved to prevent or treat MERS-CoV. The current preventative measures comprise avoidance of behaviors that lead to transmission and general health practices of handwashing, avoiding contact between the hands and the eyes and nose, and covering the nose and mouth when sneezing. Medical care comprises general supportive treatment of body organs and the use of previously known antivirals in combination with interferon (Sheahan et al., 2020). However, there is still no specific treatment produced specifically for MERS-CoV control.

The MERS-CoV genome produces four structural proteins: spike (S), membrane (M), envelope (E), and nucleocapsid (N). Fusion between the viral and cell membrane is accomplished by the two subunits of viral S protein (S1 and S2), important for completing the virus replication cycle (Bosch et al., 2003). The S1 subunit recognizes the host cell receptor, while S2 mediates the fusion process (Song et al., 2018; Hoffmann et al., 2020). Membrane fusion starts with the interaction of the HR1 and HR2 domains of S2, bringing the viral and cell membranes in proximity. Virus entry inhibitors, including attachment and fusion inhibitors, comprise an important class of antiviral drugs. Attachment inhibitors usually interfere with S binding to its receptors, which is more frequently affected by the high mutation rate (Xia et al., 2019). Fusion inhibitors interfere with the replication cycle following the attachment step and interfere with the consecutive steps of viral and cell membrane fusion. Therefore, fusion inhibitors remain an attractive strategy for discovering new antiviral drugs, especially as they affect conserved viral sequences.

Drug discovery trials against MERS-CoV comprised the production of monoclonal antibodies (Widjaja et al., 2019; Hussen et al., 2020) or repurposing previously utilized antiviral agents (Falzarano et al., 2013). In addition, a peptide sequence found in the HR2 region of wild-type MERS-CoV has been shown to have some inhibitory effect on MERS-CoV membrane fusion (Lu et al., 2014). However, this previous work has not resulted in new MERS-CoV inhibitors.

Our research group recently revealed the structure of new potential small inhibitors of the MERS-CoV fusion process by targeting cavities on the surface of HR1 (Kandeel et al., 2020). In this study, stronger fusion inhibitor peptides were designed by modification or mutation of the wild-type MERS-CoV HR2 domain, a portion of the fusion protein. The designed peptides inhibited spike protein-mediated MERS-CoV cell-cell fusion and MERS-CoV infection of cells. Thus, these peptides may be useful in preventing or treat MERS-CoV infection.

## Material and Methods

### Peptide Design

S2 HR2 was selected as the targeted peptide design site based on the findings of several studies targeting SARS-CoV, MERS-CoV, and HIV. These studies demonstrated that HR2-derived peptides were more potent than HR1 analogs (Liu et al., 2004; Lu et al., 2014; Shabane et al., 2019). In this study, the 36-amino acids wild-type HR2 peptide (Table 1, peptide 1 or SEQ ID NO: 1) was modified to obtain analogs demonstrating stronger inhibition of MERS-CoV replication (Accepted Patent, USPTO application no. 16/857136). This parent peptide was synthesized and used as a reference for comparison with the other eleven mutant peptides.

**TABLE 1:** The sequence of peptides used in this study. The sites of WT peptide mutations are underlined.

Name	Peptide sequence
Peptide 1 (WT)	SLTQINTTLLDLTYEMLSLQQVVKALNESYIDLKEL
Peptide 2	SLTQINTTLLDLTYEMLSLQQVVKALNESYIDLKHL
Peptide 3	SLTQINTTLLDLTYEMKSLQQVVKALNESYIDLKEL
Peptide 4	SLTQINWTLLDLTYEMESLQQVVKALNESYIDLKEL
Peptide 5	SLTQINWTLLDLTYEMESLQQVVKALNEYYIDLKEL
Peptide 6	SLTQINWTLLDLTYEMESLQQVVKALNEYYIDLKHL
Peptide 7	SLTQINWTLLDLTYEMESLQQVMKALNEYYIDLKHL
Peptide 8	SLTQINTTLLDLEYEMLSLQQVVKALNESYIDLKEL
Peptide 9	SLTQINTTLLDLEYEMRSLQQVVKALNESYIDLKEL
Peptide 10	SLTQINTTLLDLEYEMRSLEEVVKALNESYIDLKEL
Peptide 11	SLTQINTTLLDLEYEMRSLEEVVKKLNESYIDLKEL
Peptide 12	SLTQINTTLLDLEYEMRSLEEVVKKLNESYIDEKEL

Several computational trials were performed to optimize a new sequence related to peptide 1. Several systematic point mutations were initially generated for each amino acid in the peptide 1 sequence, providing 684 candidates with potentially improved binding energies between HR1 and HR2 (Dehouck et al., 2013). Several point mutations were then combined to yield peptides with a lower free energy of binding with HR1. Finally, eleven peptides were synthesized (Cambridge, ON, Canada, Table 1, Peptides 2-12 or SEQ ID NOs: 2-12). The peptides were purified by HPLC, and the exact mass was determined by mass spectrometry to ensure maximal purity.

### Cell Lines and Virus

Two 293FT-based reporter cell lines that constitutively express individual split proteins (DSP1-7 and DSP8-11 proteins) were used for cell-cell fusion assays (Wang et al., 2014). The cells were maintained in Dulbecco’s modified Eagle’s medium (DMEM) containing 10% fetal bovine serum (FBS) and 1 g/ml puromycin.

Vero cells were purchased from the American Type Culture Collection (ATCC, Manassas, VA, United States) for the plaque assay. The cells were maintained in DMEM containing 10% FBS (Thermo Fisher Scientific, Waltham, MA, United States), 25 mm HEPES, 100 U/ml penicillin, and 100 μg/ml streptomycin.

MERS-CoV was obtained with permission from the Korea Centers for Disease Control and Prevention (CoV/KOR/KNIH/002_05_2015, Permission No. 1-001-MER-IS-2015001). MERS-CoV amplification and quantification were performed as described previously (Kandeel et al., 2020).

### Dual Split Protein Assay to Monitor Middle East Respiratory Syndrome Coronavirus Membrane Fusion

MERS-CoV-S-mediated membrane fusion was quantitatively evaluated *via* DSP assay using 293FT cells as previously described (Yamamoto et al., 2016). The dimerization of DSP1-7 and DSP8-11 after cell-cell fusion can be quantified based on the values of fluorescence/luminescence upon formation of tight DSP complexes. The effector cells express MERS-CoV-S protein with DSP8-11, while the target cells express the MERS-CoV receptor and transmembrane serine protease 2 (TMPRSS2) with DSP1-7. The cells were grown in 10 cm culture dishes (4 × 10^6^ cells/10 ml) 24 h before the assays. Cells were treated with 6 µM EnduRen (Promega, Madison, WI, United States), a substrate for *Renilla* luciferase, for 2 h to activate EnduRen. Each peptide was dissolved in dimethyl sulfoxide (DMSO) and added to 384-well plates (Greiner Bioscience, Frickenhausen, Germany), then 50 µl of each single-cell suspension (effector and target cells) was added to the wells using a Multidrop dispenser (Thermo Fisher Scientific). After incubation at 37°C for 4 h, luciferase activity was measured using a Centro xS960 luminometer (Berthold, Germany).

### Plaque Assay After Treatment With Middle East Respiratory Syndrome Coronavirus Inhibitor Peptides and Cytotoxicity Studies

The plaque reduction assay was performed as reported previously (Kandeel et al., 2020). Briefly, Vero cells were cultivated on six-well plates for 12 h at 6 × 10^5^ cells/well. In an initial study, MERS-CoV was mixed with each peptide at a final concentration of 10 µM for 30 min at 37 °C. The mixtures of MERS-CoV and each peptide were added to Vero cells in each well and then incubated for 1 h. The supernatants were subsequently removed, and DMEM/F12 medium (Thermo Fisher Scientific) containing 0.6% oxoid agar was transferred to each well. Four days after infection, plaque formation was observed by staining with crystal violet, and plaque numbers were counted. The plaque reduction assay was repeated in a dose-dependent manner using 2-fold serially diluted samples of Peptides 4, 5, or 6 to investigate the inhibitory properties of candidate peptides. 

For the cytotoxicity assay, Vero cells (1 × 10^3^ cells/well) were incubated in 96-well plates for 12 h. The peptides were dissolved in dimethyl sulfoxide (DMSO), and cells were treated with the peptides or DMSO only for three days. The proliferation of Vero cells was analyzed using Cell Counting Kit-8 (CCK-8) (Dojindo, Rockville, MD, United States). CCK-8 solution was added to each well and incubated for 4 h at 37 °C. CCK-8 absorbance was read at 450 nm using a microplate reader (Thermo Fisher Scientific).

### Peptide Properties

Markers of peptide properties were computationally analyzed by CLC genomics software. The parameters included α-helix (residues range), α-helix%, counts of the negative charge, positive charge and non-charged residues, counts of hydrophobic, hydrophilic, other residues, and half-life in mammals.

## Results

### The Peptide Sequences

**FIGURE 1:**
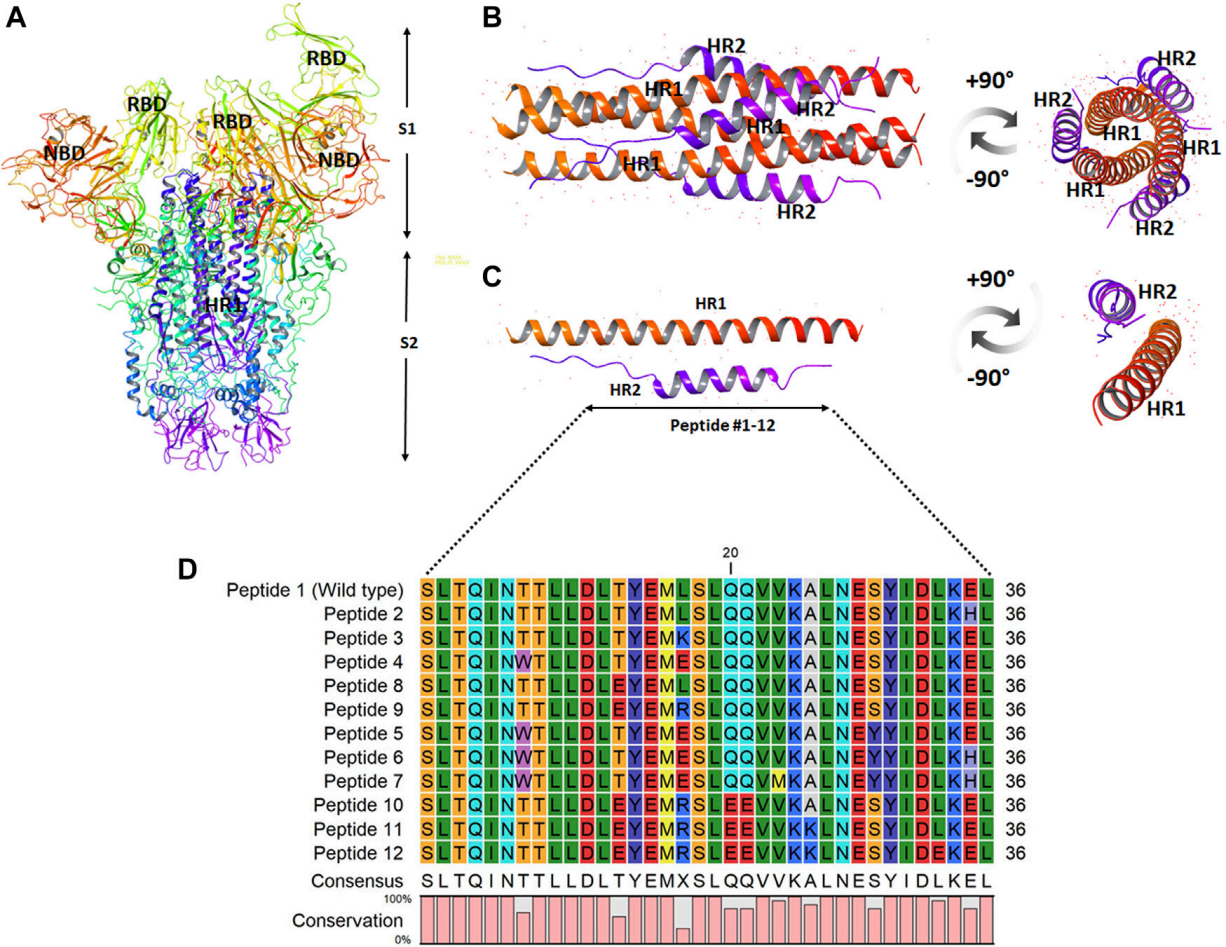
The design and sequence of peptides 1–12. **(A)** The structure of MERS-CoV spike ectodomain showing the spike S1 and S2 subunits. **(B)** The fusion core showing trimers of HR1 and HR2 in a full fusion state. **(C)** Monomers of the fusion core showing HR1-HR2 binding. The site of fusion peptide design is represented by the bidirectional arrow. **(D)** The sequence of the synthesized peptides 1–12. The color scale indicates the degree of residue conservation.

**FIGURE 2:**
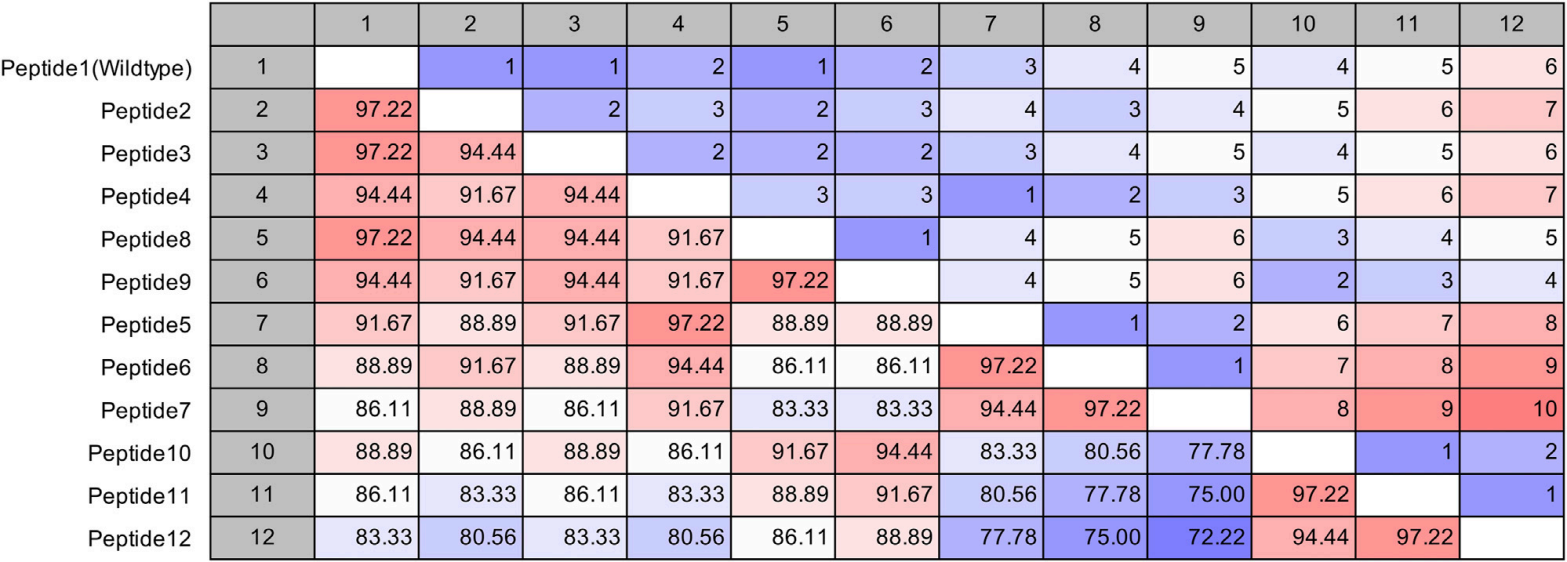
Pairwise comparison of the synthesized MERS-CoV inhibitor peptides. The upper diagonal panel shows the number of amino acid differences. The lower diagonal panel shows the identity. The color scale indicates extreme values.

The site of peptide design is shown in Figure 1. The MERS-CoV S protein is composed of two subunits S1 and S2 (Figure 1A). The S2 subunits share in the membrane fusion process, and in the full fusion state, trimers of HR1 and HR2 form the fusion core (Figure 1B). The peptides were designed to target the HR1 cavity that binds HR2 during the fusion core formation. The designed peptides were 36 amino acids long, similar to the wild type HR2 or mutated with an estimated stronger binding with HR1. The site and alignment of peptides are provided in Figures 1C,D. The peptides contained from 1 to 10 amino acid mutations, with predicted improved binding with MERS CoV HR1. The upper diagonal panel in Figure 2 shows the number of amino acid differences between each peptide. The lower diagonal panel provides the % identity, between 72.22 and 97.22%. Peptides 9, 11, and 12 showed the largest differences in amino acid composition from the wild type.

### DSP Assay for Middle East Respiratory Syndrome Coronavirus S-Mediated Cell-Cell Fusion

**FIGURE 3:**
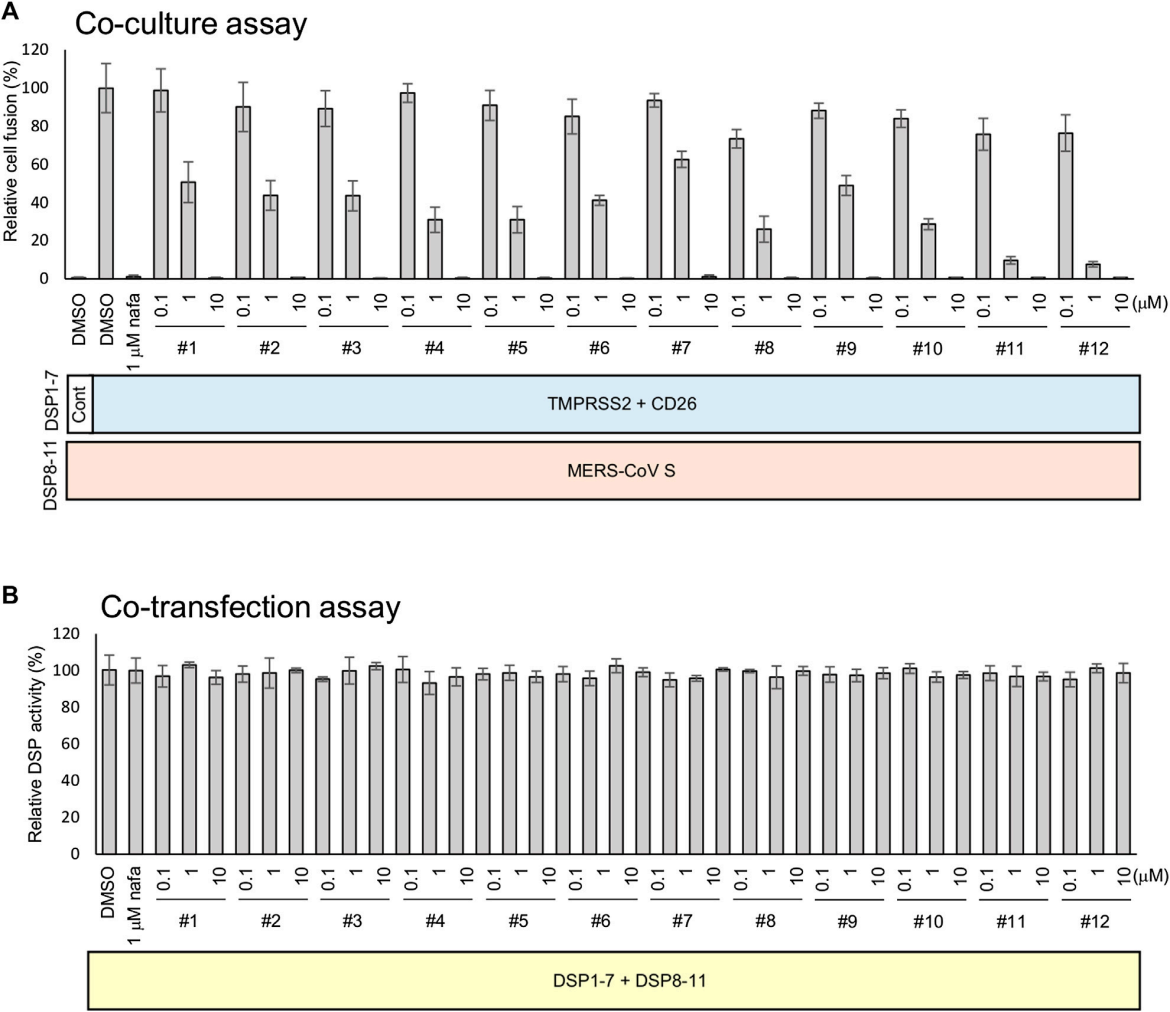
The effect of peptides on the TMPRSS2-dependent cell-cell fusion assay of MERS-CoV. **(A)** The effect of each peptide on coculture fusion using DSP as a reporter. Peptides were tested at different concentrations, and the proteins in addition to the reporters (DSPs) transduced into the effector and target cells are indicated below the graph. Nafamostat was used as an inhibitor of the TMPRSS2 pathway. The relative cell fusion was represented as the DSP value (RL activity measured in RLU) normalized to that of the control assay with DMSO alone. **(B)** The effect of each peptide on RL measurement. Each peptide was added to cells co-expressing DSP1-7 and DSP8-11 to evaluate its direct inhibitory effects on RL. The relative DSP signal is indicated on the vertical axis by representing the control value with DMSO alone to 100%.

The strength of the 12 synthesized peptides on S protein-mediated MERS-CoV fusion was evaluated as previously established (Yamamoto et al., 2016). All peptides showed significant inhibition at 10 µM (Figure 3A), yet no peptide demonstrated direct inhibition of DSP reporter activity at 10 µM (Figure 3B). The peptides inhibited MERS-CoV fusion in a dose-dependent manner. The IC50 values were from the low nanomolar to the low micromolar range (Table 2). The most effective peptides were 11 and 12 (IC50 = 0.25 µM), followed by three peptides (#5, 8, and 10) with IC50 = 0.3–0.6 µM.

**TABLE 2:** EC50 values of peptides determined by cell-cell fusion assay.

Peptide	EC50 (µM)
1 (SEQ. ID NO:1)	1.3
2 (SEQ. ID NO:2)	0.94
3 (SEQ. ID NO:3)	0.93
4 (SEQ. ID NO:4)	1.7
5 (SEQ. ID NO:5)	0.58
6 (SEQ. ID NO:6)	0.82
7 (SEQ. ID NO:7)	2.3
8(SEQ. ID NO:8)	0.34
9 (SEQ. ID NO:9)	1.2
10 (SEQ. ID NO:10)	0.48
11 (SEQ. ID NO:11)	0.25
12(SEQ. ID NO:12)	0.25

### Plaque Inhibition Assay

**FIGURE 4:**
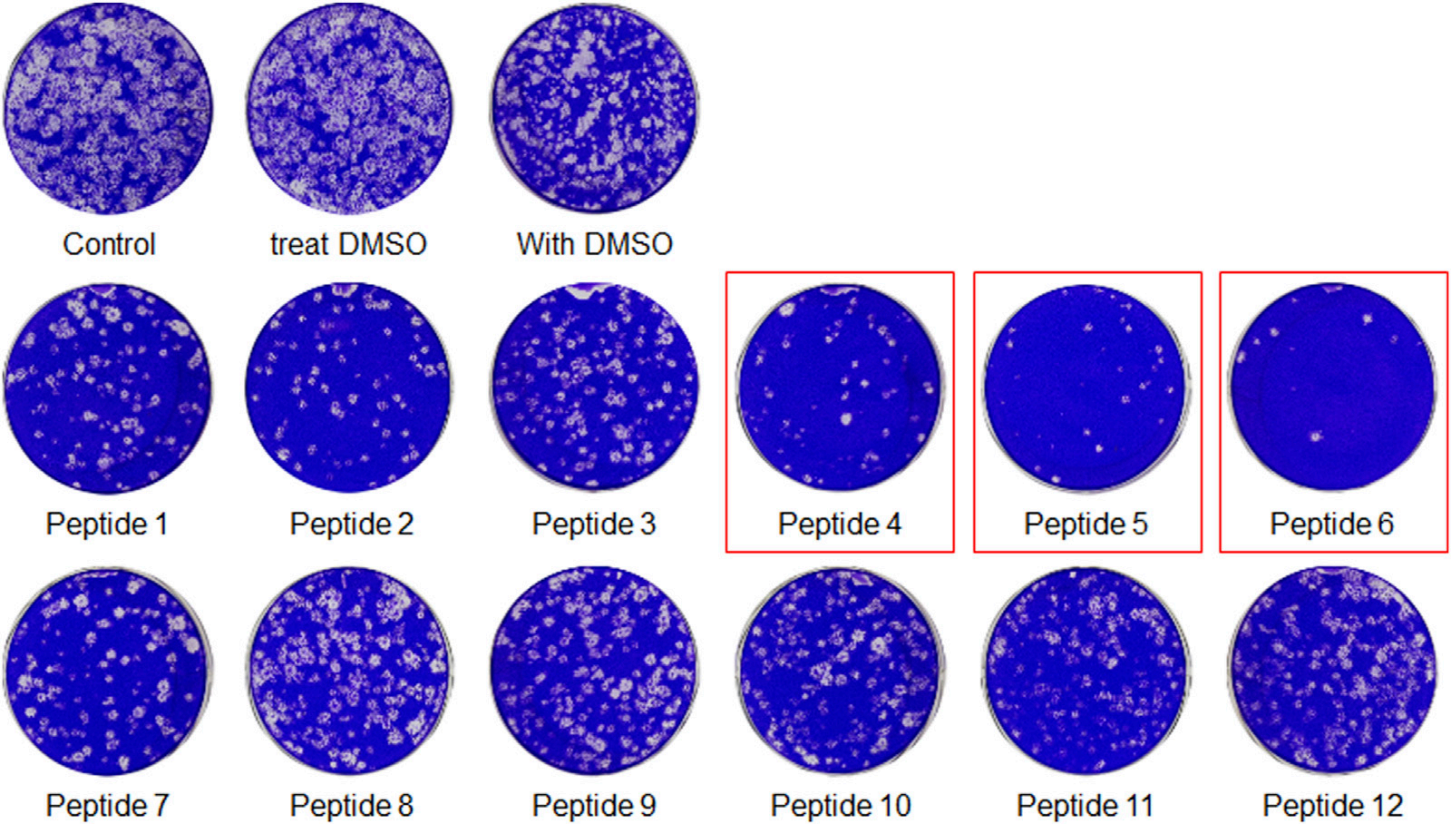
The plaque formation assay for MERS-CoV inhibitor peptides. MERS-CoV was pre-incubated with 10 µM of each peptide for 30 min at 37°C. The mixture of the virus and each peptide was added to the Vero cells and incubated for 1 h. After the incubation, the medium was replaced with DMEM/F12 containing 0.6% oxoid agar. The plaques were stained with crystal violet 4 days after infection. Plaque number was quantified and relative production of viral particles is shown, with virus production of a DMSO-treated control representing 100%.

**FIGURE 5:**
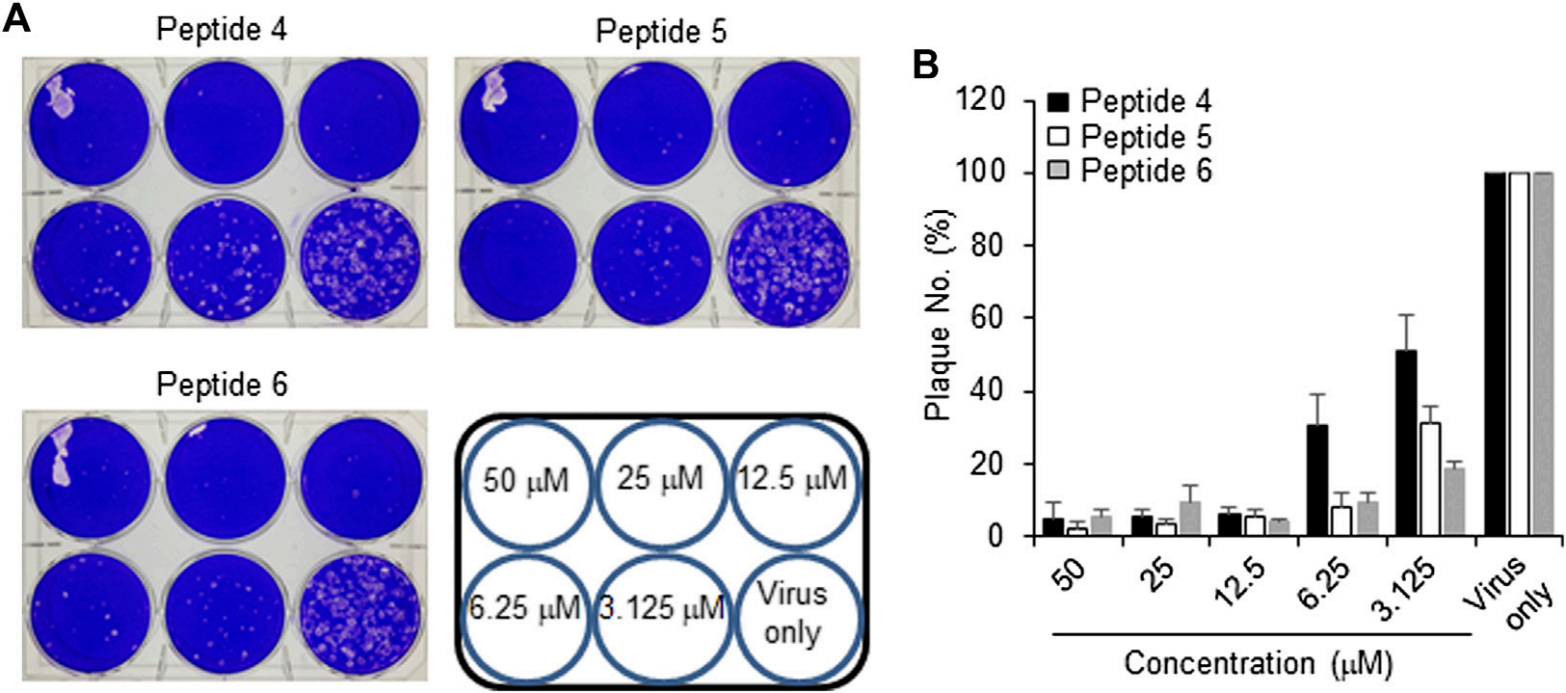
Effect of inhibitor peptides on MERS-CoV infection. MERS-CoV was pre-incubated with two-fold serially diluted peptide 4, 5 or 6 (*n* = 3) for 30 min at 37°C. Vero cells were treated with the mixture of the virus and each peptide and then incubated for 4 days in DMEM/F12 containing 0.6% oxoid agar. The plaques were observed by staining with crystal violet and counted **(A)** A representative picture showing the plaque reduction assay **(B)** Quantification of the plaque reduction assay against MERS-CoV after treatment with each peptide.

The peptides were initially screened at 10 µM concentration in MERS-CoV plaque assay (Figure 4). All peptides inhibited MERS-CoV plaque formation. Based on the degree of plaque inhibition, three peptide classes were identified: strong, moderate, and weak inhibitors. Three peptides (Bosch et al., 2003; Bosch et al., 2003; Song et al., 2018; Sheahan et al., 2020) strongly reduced MERS-CoV plaque formation by more than 95%. Five other peptides, 2, 7, 10, 11, and 12, showed 69–74% inhibition of plaque formation. Peptides 1 and 3 showed a 64% decrease in MERS CoV replication. To quantitatively evaluate the effect of peptides on viral replication, the plaque assay was repeated at different concentrations of peptides 4, 5, and 6 using two-fold serially diluted concentration from 50 µM. As shown in Figure 5, MERS-CoV plaque formation decreased in a concentration-dependent manner.

### Cytotoxicity and Viability

**FIGURE 6:**
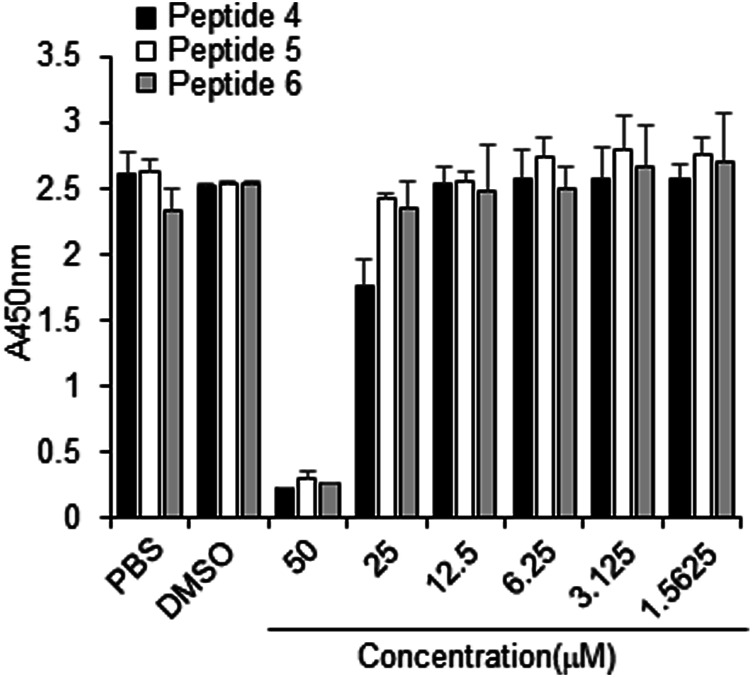
Effect of peptides 4, 5, and 6 on the proliferation of Vero cells. Peptides (100 µM) were dissolved in 10% DMSO, and then the peptides were two-fold serially diluted in PBS. Vero cells were treated with PBS, 1% DMSO, or indicated peptide concentrations for 3 days, followed by the CCK-8 assay.

The cytotoxicity of peptides 4, 5 or 6, or DMSO (control) was examined in Vero cells for 3 days using concentrations up to 50 µM. No cytotoxicity was observed at or below 10 µM for any tested peptide (Figure 6). Thus, peptides 4, 5, and 6 have a safe cellular profile without cytotoxicity.

### Molecular Properties of Peptides

The designed peptides computationally demonstrated stronger binding with MERS-CoV HR1. The peptides showed 1–6 mutations relative to the wild type. Despite the mutations, all peptides maintained high α-helix content with a constant residues range of 2–34 and a high helix content rate of 91.7% (Table 3). The negative and positive charged residues were in the ranges 4–9 and 2–4, respectively. Most of the peptide compositions were from the non-charged regions (23–30 residues).

**TABLE 3:** The protein structure statistics of the MERS CoV inhibitor peptides.

ID	α-helix (residues range)	α-helix %	Counts of residues			Frequency of residues			
			Negative charge	Positive charge	Non-charged	Hydrophobic	Hydrophilic	Other	Half-life in mammals (h)
# 1	2–34	91.7	5	2	29	15	14	7	1.9
# 2	2–34	91.7	4	2	30	15	14	7	1.9
# 3	2–34	91.7	5	3	28	14	14	8	1.9
# 4	2–34	91.7	6	2	28	15	13	8	1.9
# 5	2–34	91.7	6	2	28	15	13	8	1.9
# 6	2–34	91.7	5	2	29	15	13	8	1.9
# 7	2–34	91.7	5	2	29	15	13	8	1.9
# 8	2–34	91.7	6	2	28	15	13	8	1.9
# 9	2–34	91.7	6	3	27	14	13	9	1.9
# 10	2–34	91.7	8	3	25	14	11	11	1.9
# 11	2–34	91.7	8	4	24	13	11	12	1.9
# 12	2–34	91.7	9	4	23	12	11	13	1.9

## Discussion

Despite the emerging and fatal nature of MERS-CoV, structure-based drug discovery studies to combat this virus are very limited. Computational studies have substantially contributed to the drug discovery process, especially through lead identification and optimization (Koutsoukas et al., 2011). Small molecule inhibitors against several MERS-CoV targets were provided to the research community (Xia et al., 2014; Kumar et al., 2016; Galasiti Kankanamalage et al., 2018). Recently, we designed the first generation of small chemical MERS-CoV fusion inhibitor molecules (Kandeel et al., 2020). In addition, several designed peptides were proven efficient in inhibiting SARS-CoV-2 replication (Kandeel et al., 2021). Complementing these efforts were short peptides demonstrating stronger inhibition in the nanomolar range than the previously provided small molecules, which showed inhibition of MERS-CoV fusion in the low micromolar range. The discovery of new drugs and the design of novel vaccines are the two most powerful tools for controlling viral diseases. In this context, fusion inhibitors were a promising class of antiviral drugs extensively studied in treating influenza virus (Kadam et al., 2017), dengue virus (De La Guardia & Lleonart, 2014), respiratory syncytial virus (Feng et al., 2015), African swine fever virus (Hakobyan et al., 2018), measles virus (Welsch et al., 2013), HIV (Jing et al., 2002), SARS-CoV (Liu et al., 2004; Sainz et al., 2006; Chu et al., 2008; Liu et al., 2009), and MERS-CoV (Lu et al., 2014).

Recently, peptide-based therapeutics have contributed enormously in terms of improved stability and systemic bioavailability. The advantages of developing peptide drugs are their predictable and optimizable absorption distribution, metabolism, and excretion properties (Fosgerau & Hoffmann, 2015). The premise of using peptide drugs as fusion inhibitors has successfully delivered a clinical drug for treating HIV (Ding et al., 2017). Cell membranes and body barrier penetration have been improved using optimized amphipathic and α-helical peptides (Stalmans et al., 2015). More robust tools have been used to improve the oral bioavailability of therapeutic peptides, e.g., oral application of insulin-polyarginine conjugates (Morishita et al., 2007). Inhalable peptides were also used with promising results in treating pulmonary and systemic diseases (Greene et al., 2020; Waxman et al., 2021). These technologies offer a promising future for peptide drug development.

The spike-activated CoV entry into cells was proven to be by either direct membrane fusion or endocytosis (Seyedpour et al., 2020). In these entry paths, the viral spike must be activated by cellular proteases such as TMPRSS2 and cathepsin L for membrane fusion and endocytosis, respectively. The expression of TMPRSS2 in human tissues was found to be specific for each cell type (Sungnak et al., 2020; Ziegler et al., 2020). In mouse models, the spread of SARS-CoV and MERS-CoV was limited by a TMPRSS2-knockout (Iwata-Yoshikawa et al., 2019). In this study, the S-mediated dual split cell-cell fusion in 293FT cells was dependent on TMPRSS2 expression. Thus, these findings suggest that the peptides may inhibit the TMPRSS2-dependent plasma membrane fusion of MERS-CoV. The 293FT cell lines utilized were overexpressing TMPRSS2. As Vero cells lack expression of TMPRSS2, viruses enter by the endocytic pathway (Hoffmann et al., 2020). Therefore, the observed effect of peptides suggests the potential dual action of the peptides on both membrane fusion and endocytosis paths.

The selection of a peptide 36 amino acids in length was based on previous cell-cell fusion assays (Lu et al., 2014). In this study, the 36-mer peptide inhibited MERS-CoV S-mediated cell-cell fusion in the low nanomolar to the low micromolar range. In the drug discovery process, the binding of the fragment to its target is usually in the millimolar to micromolar range (Li, 2020). Current clinically used drugs operate within the micromolar range (Lomenick et al., 2009). Weak binding affinity has been defined as within the millimolar or high micromolar range (Li, 2020). Novel antiviral compounds with IC50 values in the low micromolar range are expected to be promising lead structures (Liu et al., 2004). One example is enfuvirtide, an FDA-approved fusion inhibitor of HIV-1 that showed extended IC50 values from 10 ng/ml to 7 μg/ml (2  nm–7 µM concentrations) (Greenberg, 2007). Based on these data, the reported peptides in our work could have clinical implications in controlling MERS-CoV infection.

**FIGURE 7:**
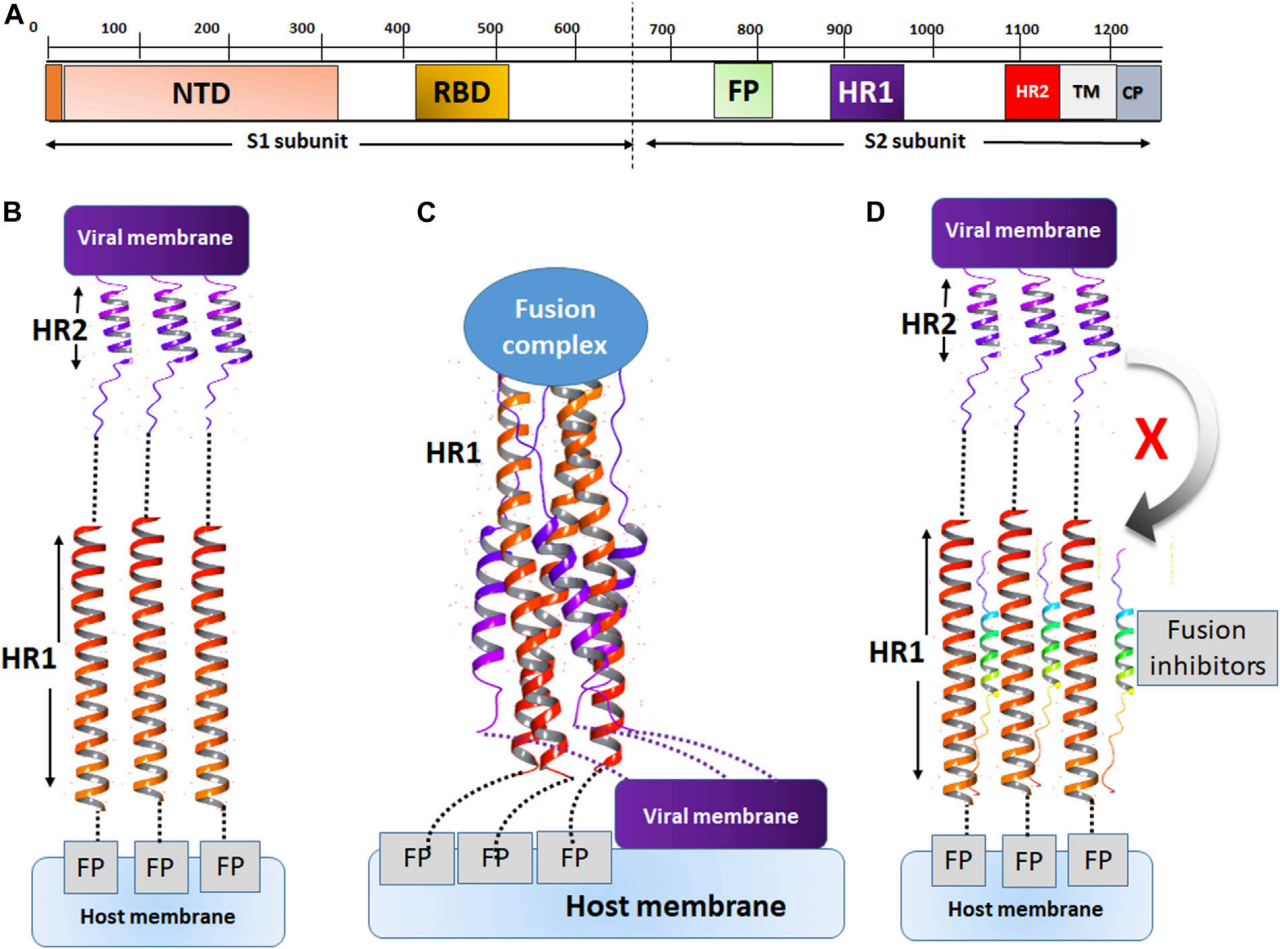
The mechanism of action of fusion inhibitors. **(A)** The composition of MERS-CoV S protein. The S1 subunit contains the nucleotide binding domain (NBD) and the receptor binding domain (RBD). The S2 subunit contains the fusion protein (FP), HR1, HR2, the transmembrane domain (TM) and the cytoplasmic domain (CP). **(B)** The prefusion conformation. HR2 assists in the fusion between the viral and host cell membranes. **(C)** The conformation of fusion state. The viral and cell membranes move in close position and membrane fusion occurs. **(D)** The fusion inhibitor peptides bind to HR2 and prevent the recognition of HR2 onto its binding sites on HR1.

The mechanism of action of fusion inhibitors is provided in Figure 7. The MERS-CoV S protein is composed of the S1 and S2 subunits (Figure 7A). The S1 subunit recognizes the host cell membrane through the receptor binding domain (RBD). After cleavage by host proteases, the fusion protein (FP) of S2 binds the host cell membrane in the presence of viral proteins across the host membrane (TM) and an intracellular portion called the cytoplasmic domain (CP). For fusion to occur, the host and viral membranes come in close apposition via the interaction of HR1 and HR2 (Figure 7C). The peptides developed in this study were designed to target a surface cavity on HR1, thus competing with HR2 for their binding sites on HR1 and preventing the viral fusion process.

Recent reports indicate that the improved α–helicity of HR2 in SARS-CoV-2 produced stronger fusion complexes with HR1, compared with SARS-CoV (Zhu et al., 2020). In addition, fusion peptides with enhanced α-helical content have been associated with higher antiviral efficacy (Sainz et al., 2006). In this study, all peptides showed similar α-helical content of 91.7% (Table 3). The strong MERS-CoV S-mediated inhibition we observed in the cell-cell fusion assays might be attributed to the combination of the improved energy of binding as well as the improved peptide α–helicity. Interestingly, a CoV HR2 derived peptide (HKU4-HR2P2) from a bat was able to inhibit MERS-CoV-mediated cell-cell fusion at a concentration of 380 nm (Xia et al., 2019). In another study, the EC50 of strong peptide P1 against MERS-CoV infection was 3.013 µM (Gao et al., 2013). Multiple systematic mutations with charged residues in MERS-CoV HR2 lead to the discovery of potent peptides that inhibited cell-cell fusion with IC50 values of 550–930 nm. Based on these data, the present peptides demonstrated notable potency by inhibiting cell-cell fusion at 250 nm concentration.

In conclusion, in the search for new anti-MERS-CoV agents, a structure-based approach was used to develop a MERS-CoV fusion protein inhibitor. A set of peptides were provided with potent inhibition of cell-cell fusion and MERS CoV replication. These peptides may be effective in optimizing specific anti-MERS CoV agents.

## Data Availability

The original contributions presented in the study are included in the article/Supplementary Material, further inquiries can be directed to the corresponding authors.
